# Guidewire-First Rescue Versus Direct Suprapubic Cystostomy After Failed Urethral Catheterisation: A Propensity-Weighted Cohort Study

**DOI:** 10.3390/jcm15207792

**Published:** 2026-10-09

**Authors:** Ahmet Şanlı, Serhat Göçer, Hasan Samet Güngör

**Affiliations:** 1 Department of Urology, Karamanoğlu Mehmetbey University Training and Research Hospital, Üniversite Mahallesi, Şehit Ömer Halis Demir Caddesi No: 7/1, 70100 Karaman, Türkiye; 2 Department of Urology, Tekirdağ Dr. İsmail Fehmi Cumalıoğlu City Hospital, İstiklal Mahallesi, Yemen Sokak No: 13, 59030 Tekirdağ, Türkiye; serhatemir2012@gmail.com; 3 Department of Urology, University of Health Sciences, Ümraniye Training and Research Hospital, Elmalıkent Mahallesi, Adem Yavuz Caddesi No: 1, 34760 Istanbul, Türkiye; drsametgngr@gmail.com

**Keywords:** urinary retention, urinary catheterisation, suprapubic cystostomy, guidewire, emergency urology, propensity score, difficult urinary catheterisation

## Abstract

**Background/Objectives:** When urethral catheterisation fails in acute urinary retention, clinicians may pass a guidewire or go directly to suprapubic cystostomy. No published series compares the two after a failed attempt, and comparative trials enrol men in whom either route remains available. We compared the two. **Methods:** We studied 352 consecutive men at one centre from 2019 to 2025: 246 went directly to cystostomy, 106 to an ultrasound-confirmed bedside guidewire attempt. Time zero was the documented failure of catheterisation, and patients were analysed by the strategy first attempted. The primary outcome, overall strategy-associated complications, was any new Clavien–Dindo grade II or higher complication within 30 days, whichever route produced it; conditions present at time zero did not count. Overlap weighting on 19 covariates gave risk differences with operator-level bootstrap intervals. Follow-up used the hospital and national health records for every patient. **Results:** The guidewire attempt placed a catheter in 84.9% of men (95% confidence interval [CI] 76.9 to 90.5). After weighting, overall strategy-associated complications affected 17.6% of the cystostomy arm and 5.3% of the guidewire-first arm (−12.2 points, 95% CI −19.0 to −5.5). Grade III or higher events occurred in 7.7% (19/246) of the cystostomy arm and none of the guidewire-first arm. Shared complications, those either route could produce, affected 9.5% and 5.3%, respectively, a difference of −4.2 (−11.2 to 3.8) after weighting and −0.3 (−6.0 to 5.4) when unweighted. **Conclusions:** An ultrasound-confirmed bedside guidewire attempt was associated with fewer overall strategy-associated complications at 30 days. For shared complications the difference was smaller and no longer excluded zero. Ascertainment was record-based and asymmetric: cystostomy-specific harms are readily recorded, whereas urethral harms such as false passage or later stricture were not captured, and time to definitive drainage was not measured. These findings describe initial rescue strategies and do not establish that either procedure is intrinsically safer.

## 1. Introduction

Acute urinary retention is a common urological emergency [1,2]. The first attempt at a urethral catheter usually succeeds. When it does not, the patient still needs draining, and the clinician needs a second move. Of 841 urology catheter consultations at one centre, 181 were classified as difficult, a fifth of the total [3].

Failure at the first attempt is not a neutral event. Traumatic catheterisation occurs in 6.7 per 1000 catheters inserted in one prospective series; 81% of the injured men sustain a complication of Clavien–Dindo grade II or above, and their inpatient stay lengthens by a mean of 9.4 days [4]. Long-term follow-up of that cohort shows stricture disease and repeated dilatation years later [5], while a separate series at another centre records the early sequelae and the hospital resources they consume [6]. Of 37 iatrogenic catheterisation injuries recorded in one prospective series, nine patients (24%) required an indwelling suprapubic catheter [4]. Among the men with catheterisation injuries, therefore, a suprapubic tube is not only the means of rescue but often the drainage method that persists.

The suprapubic route carries its own hazards, and they are better quantified than the urethral ones. A national audit recorded 11,473 suprapubic insertions; the meta-analysis published with it found bowel injury in 0.7%, and the audit recommended counselling low-risk patients with a figure below 0.25% [7]. National guidance on suprapubic-catheter practice has been revised in response [8]. The choice at the bedside is therefore between two procedures that can both harm, and the harms are of different kinds.

Guidance acknowledges the alternative without grading it. The European Association of Urology states that cystoscopic guidewire-led catheter insertion may prevent urethral trauma in difficult catheterisation cases [9]. That statement sits in a narrative subsection on epidemiology and aetiology and specifies a cystoscopic technique; whether the same holds for a guidewire passed without a cystoscope is not addressed. It is not carried into the guideline’s evidence summary or its recommendation table, which on catheterisation address training alone. The same guidance puts the incidence of male urethral injury during transurethral catheterisation at 6.2 to 13.4 per 1000 catheters inserted.

Published protocols for difficult catheterisation describe escalation pathways rather than a comparison of routes [10,11], and the evidence on wire-led rescue is a set of device and technique cohorts. An early report described the Seldinger technique as a gentler alternative to suprapubic puncture [12]. A prospective series later placed a catheter in 107 of 115 difficult catheterisations, performed by doctors without urology training [13]. Purpose-built devices with an integrated wire or an endoscopic guide have been evaluated in their own cohorts [14,15,16]. Fluoroscopic placement rescues cases in which blind or cystoscopy-assisted catheterisation has failed [17,18]. Only one of these device and imaging series carried a comparison group, and it set a purpose-built device against standard catheterisation in men whose first attempt had not yet failed [14].

Comparative evidence for the two routes exists and answers a different question. In a Cochrane review of 42 randomised and quasi-randomised trials, 25 compared the two routes; the review concluded that suprapubic catheterisation reduced asymptomatic bacteriuria, recatheterisation and pain relative to an indwelling urethral catheter, on low to very low-quality evidence, while the evidence on symptomatic urinary tract infection was inconclusive [19]. Those trials enrolled patients in whom either route was available and the choice was a preference. Once the urethral attempt has failed, the choice becomes a rescue decision, and the trials do not speak to it.

What is missing is a comparison of the two rescue strategies against each other, and an outcome set that can carry such a comparison. The evidence gap lies directly beneath a common emergency urology decision. The severe harms belong to one route only: a cystostomy can be malpositioned or its tract lost, and neither event is available to the other route. Milder harms are shared. A strategy comparison must therefore state explicitly whether route-specific events are part of the estimand.

We compared the two rescue strategies directly. The cohort was 352 consecutive men whose urethral catheterisation had failed. Route-specific events were kept in the primary outcome, overall strategy-associated complications, because avoiding them is part of what a guidewire-first strategy does. A second outcome, shared complications, held the same severity threshold and removed them, so the comparison can be read both with the device and without it.

## 2. Materials and Methods

### 2.1. Design, Setting and Participants

The question is which first move should follow a failed catheterisation, so the design compares the two moves as they were actually used. This was a retrospective cohort study at a single tertiary centre, the University of Health Sciences Ümraniye Training and Research Hospital, Istanbul. Patients were identified from the hospital information system, in which every urology consultation recorded over the study period was reviewed: 10,755 consultations in all. The entry definition was acute urinary retention with a documented failed urethral catheterisation before urology review. Failure was recorded by the admitting team as at least one unsuccessful attempt at standard catheterisation. Only men were eligible: sex was taken from the hospital record, and the rescue strategies compared here are performed on the male urethra, so no female patient entered the cohort.

**Figure 1:**
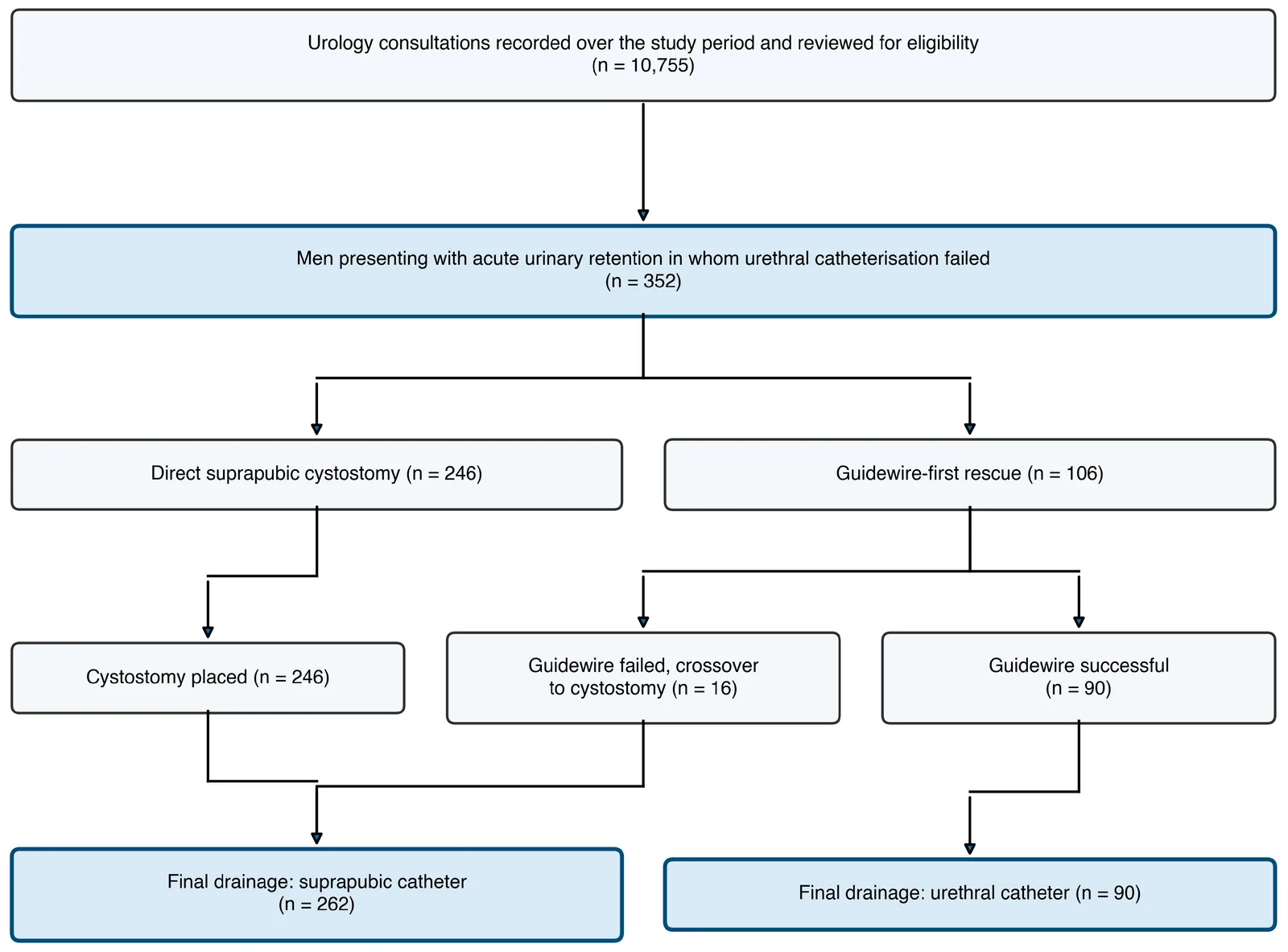
Study flow, from the urology consultations reviewed for eligibility to the drainage each man left with. Men presenting with acute urinary retention in whom urethral catheterisation failed are grouped by the strategy first attempted. The sixteen men whose guidewire attempt failed join the direct-cystostomy patients in the final suprapubic row and remain in the guidewire-first arm throughout the analysis.

All 352 records meeting this definition from January 2019 through December 2025 were included; no further eligibility criterion was applied, and no patient was removed for missing data. Figure 1 shows the flow of patients through the study. No covariate in the propensity model had a missing value. The laboratory covariates—haemoglobin, platelet count, international normalised ratio (INR) and creatinine—are part of the routine panel drawn at presentation in acute retention; height and weight are entered on the emergency department observation form, and retention volume is read from the bladder ultrasound performed before drainage, which is why these covariates are complete for every patient rather than selectively recorded.

Both rescue strategies ran in parallel throughout. The share of men taken to a guidewire attempt did not trend across calendar years (odds ratio [OR] 1.04, 95% confidence interval [CI] 0.92 to 1.17, *p* = 0.56), which matters because a two-strategy cohort assembled over seven years usually reflects a technique that was adopted partway through. Fifteen urologists performed the procedures. Records were abstracted from the hospital record by a single reviewer.

### 2.2. The Two Strategies

Neither strategy was protocolised, and the choice rested with the attending urologist. Whether the reviewing urologist repeated a standard catheter attempt before escalating was not recorded. The comparison is therefore between the moves that followed the admitting team’s failure, not between a urologist’s first and second choices.

In the guidewire-first strategy, a 0.035- to 0.038-inch hydrophilic guidewire was advanced through the external urethral meatus into the bladder. Its position was accepted when the wire was seen coiling within the bladder on ultrasound. A 16 to 18 Fr latex or silicone Foley catheter was then advanced over the wire through a fine opening made at the catheter tip, as a standard catheter carries no channel for a guidewire. Cystoscopy formed no part of this technique; a field in the source record that flagged cystoscopic assistance did not describe the procedure performed here and was withdrawn before analysis. The number of passes attempted before the wire was abandoned and the point at which the attempt was declared failed were not standardised and are not retrievable from the record. Crossover to cystostomy followed the operator’s judgement at the bedside.

The cystostomy technique was uniform. It was performed under ultrasound guidance, with the bladder confirmed full before puncture, using a single-puncture trocar set with a 12 to 16 Fr catheter. A Seldinger over-the-wire technique for suprapubic placement was not in use at the centre during the study period. Both strategies were therefore performed under ultrasound guidance, and the comparison is not one of imaging against no imaging. Ultrasound was used in every cystostomy and in every guidewire attempt without exception, including cystostomies that followed a failed attempt; this was invariable practice at the centre throughout the study period.

The guidewire attempt was performed under urethral anaesthetic gel and the cystostomy under infiltrative local anaesthesia of the skin and fascia. No sedation was used in either. Neither carried antibiotic prophylaxis, so prophylaxis cannot differ between the arms. Neither procedure appears among the interventions covered by the European Association of Urology guideline on urological infections in its periprocedural prophylaxis recommendations, and for urethral catheterisation that guideline recommends against prophylactic antimicrobials to prevent catheter-associated infection [20].

### 2.3. Time Zero and Treatment Assignment

Time zero was fixed by an event, not a decision. It was the moment urethral catheterisation was documented as failed, and Table S1 sets out the target trial this analysis is aligned to and the points at which the analysis departs from that trial [21]. Treatment assignment was not randomised; it was adjusted for using overlap weighting under an assumption of conditional exchangeability given the measured covariates. Each man was assigned to the strategy first attempted after that moment, and whatever followed was analysed under that assignment. The 16 men whose guidewire attempt failed and who then received a cystostomy therefore remain in the guidewire-first group. Analysing them under the strategy they ended with would condition on the outcome of the attempt, which is the quantity the study is trying to estimate.

### 2.4. Outcomes

The primary outcome, termed overall strategy-associated complications, was any new complication of Clavien–Dindo grade II or above within 30 days [22], counted under the strategy first attempted and with every event counted regardless of which route produced it. New means arising after time zero: a condition a man already carried when the rescue began is not a complication of the rescue, and the one endpoint component he can carry in is a febrile urinary tract infection. Route-specific events remain in the outcome. A cystostomy that is never placed cannot be malpositioned, and avoiding that exposure is part of what a guidewire-first strategy does. The crossover patients keep the guidewire-first arm exposed to cystostomy complications, so the comparison is not structurally fixed.

Each event was defined before the outcome was assembled. A febrile urinary tract infection was recorded when a documented temperature above 38 °C accompanied treatment for a presumed urinary source. Visible haematuria was blood in the urine seen without a microscope, and catheter blockage was a cessation of drainage that required irrigation or exchange. Transfusion was given for symptomatic anaemia rather than at a fixed haemoglobin threshold. Malposition was recorded when the suprapubic catheter stopped draining and ultrasound showed it outside the bladder; loss of the tract was when the catheter left the tract and could not be replaced through it. Both required a reintervention under local anaesthesia.

New meatal bleeding was counted only in men whose urethra was not already bleeding at time zero. Blood at the meatus tracked the attempts that preceded the rescue, so the unrestricted field could not carry a comparison. The restricted field was checked against the file in both arms by the same criterion, so new meatal bleeding can be compared between them. Readmission within 30 days is not reported as a separate outcome because the field was recorded only when a grade II or above event was already present and therefore repeats the outcome rather than adding to it.

Three of these need comment in relation to published standards. The European Association of Urology defines catheter-associated infection microbiologically, by growth of 10^3^ colony-forming units per millilitre or more [20]. Urine culture results were not retrievable for this cohort, so only the clinical limb of that definition could be applied. No guideline defines blockage, malposition or loss of the tract operationally, and the definitions above are the ones this centre used. Transfusion followed clinical judgement rather than a fixed haemoglobin threshold, so this cohort should not be described as meeting the restrictive threshold of the 2023 AABB guidance [23]. The men transfused presented anaemic: baseline haemoglobin 8.9 (interquartile range [IQR] 8.7–9.4) g/dL, falling by 2.8 (IQR 2.7–3.3) g/dL to a nadir of 6.1 (IQR 5.9–6.4) g/dL, and 12 of 13 fell below 7 g/dL, so practice was in fact close to a restrictive standard.

Grade followed the event rather than a judgement. A transfusion, or a febrile infection treated with antibiotics, is grade II. A malpositioned tube or a lost tract is grade IIIa, each having required a reintervention under local anaesthesia, and the single bowel injury requiring laparotomy is grade IIIb. Visible haematuria and catheter blockage are grade I unless they led to one of those. What a patient carries is therefore fixed by the events recorded for him rather than by a separate severity judgement. The map has a ceiling at the event level: no shared event can rise above grade II however severe its course, so a worsening urosepsis carries the same grade as one that settles on antibiotics. Vital status at 30 days was checked for every patient in the national record, and no death occurred in either arm, so grade V is an ascertained zero. Organ support was not separately abstracted, so grade IV rests on the absence of a recorded event rather than on a separate review.

A secondary outcome, termed shared complications, held the same grade threshold and removed the route-specific events. A patient counted if he had a new febrile urinary tract infection or a transfusion. These are the two shared events that reach grade II on their own; visible haematuria and catheter blockage are graded I, so neither can carry a patient into the outcome by itself.

Holding the threshold fixed matters, because an outcome that also drops the grade requirement changes two things at once and cannot isolate either. An exploratory any-grade composite of four shared events is also reported: febrile infection, transfusion, visible haematuria and catheter blockage. That composite changes a third thing: it counts every recorded febrile infection, including one present at time zero, so it is not comparable with the primary outcome on that component either. Route-specific events were tabulated by the route that can produce them, as description rather than comparison.

Febrile urinary tract infection is the one component of the outcome a man can bring with him. Among the men with a recorded febrile urinary tract infection, the infection was already present at time zero in 74.1% (20/27) of the cystostomy arm and 42.9% (3/7) of the guidewire-first arm. The rule stated above therefore excludes them, as it excludes blood at the meatus in men whose urethra was already bleeding: an event a patient already has is not a complication of what was done to him.

A wider composite is also kept as a sensitivity analysis, counting every recorded grade II or above event, including prevalent infection, and reported alongside. It is the outcome an audit of the record produces without asking when the condition began. The wider composite, unlike the primary outcome, was not specified in advance; neither it nor the shared-complications outcome is confirmatory, and Section 3.3 and Section 3.4 give each. A further sensitivity outcome counts every recorded complication of any grade, route-specific events included; it is the least specific outcome reported and is shown for completeness.

Composition of the primary outcome by highest grade and event type is given in Table S2. Every patient was followed for the 30 days from time zero in two record systems: this hospital’s information system and e-Nabız, the national personal health record maintained by the Ministry of Health of Türkiye. Ascertainment therefore extended beyond this institution. It remains record-based, so an event that generated no reported encounter would not appear.

Guidewire success was defined as a urethral catheter in place at the end of the attempt. Markers of guidewire failure within the guidewire-first arm were described separately; none of the markers was specified in advance.

### 2.5. Statistical Analysis

Treatment assignment was modelled with logistic regression on 19 baseline covariates. Demography and physiology contributed age, body-mass index, retention volume, creatinine, haemoglobin and platelet count. Lower urinary tract history contributed known benign prostatic hyperplasia, known urethral stricture, suspected bladder-neck contracture, prior transurethral or laser prostatectomy, prior urethral surgery, prior difficult catheterisation and prior lower abdominal surgery. The state at presentation contributed urinary tract infection, urethral bleeding before rescue, diabetes, oral anticoagulant use, antiplatelet use and the international normalised ratio. Anticoagulants and antiplatelet agents were entered as separate terms because their clinical effects and imbalance between arms differed. The model contained 19 main-effect terms for 106 guidewire-first patients; this limited the scope for flexible parameterisation and motivated the model-size checks described in the Supplementary Methods S1.

**Figure 2:**
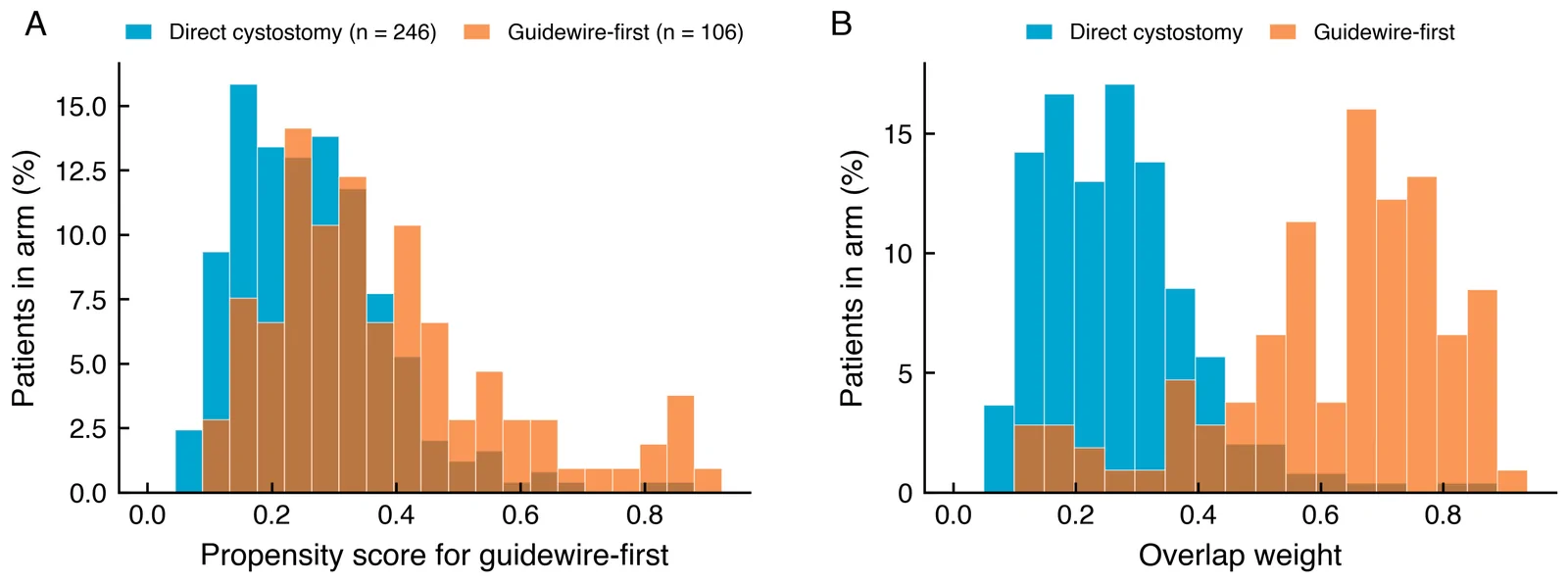
Propensity overlap and weight distribution for the primary analysis. (**A**) Distribution of the estimated propensity score for guidewire-first in each arm, as a percentage of patients within that arm so the two can be compared despite their different sizes. (**B**) Distribution of the overlap weights on the same scale. The largest single weight carries 0.7% of the total weight. Overlap weighting equalises the weighted mean of every covariate entered in the model, by construction, so balance is not plotted; Table S3 reports the diagnostics the weights do not fix. Where the two distributions overlap, the blue and orange bars combine to brown.

Continuous covariates entered the score linearly. Overlap weights were applied to the fitted scores [24]. Overlap weighting targets the population in which both strategies are genuinely used, that is, patients whose covariate profiles appear in both arms, and it equalises the weighted mean of every covariate entered in the model. The estimate therefore applies to that overlap population rather than to the whole cohort. That equality is an algebraic property of the weights rather than a finding about these data. It holds for the terms entered in the model and not for the whole distribution of a covariate, and Figure 2 makes this clear. Balance was therefore also assessed beyond those means using variance ratios for the continuous covariates, which weighting does not equalise, and standardised differences for three baseline variables that never entered the model [25]. Table S3 reports both and notes which of the three the model terms determine anyway.

The estimand was an absolute risk difference. It compares the risk of each outcome between strategies. Confidence intervals are percentile intervals from 2000 bootstrap resamples that refitted the propensity model within each resample, since treating the weights as fixed understates the variance. Resampling was at the level of the operator rather than the patient because fifteen surgeons choosing between two strategies is a clustered design, whatever a heterogeneity test shows. Fifteen clusters is few for a cluster bootstrap, so the analysis was also refitted omitting each operator in turn, and Table S4 reports strategy use by operator. A further sensitivity analysis adds operator fixed effects to the propensity model, so that the operator is treated as a possible confounder and not only as a cluster.

Sensitivity analyses re-estimated the primary and the shared-complications contrasts under stabilised inverse-probability weights, a doubly robust estimator, a propensity model containing calendar year, and a clinically restricted propensity model. Their specifications, including the events-per-parameter ratio of the doubly robust outcome model, are given in the Supplementary Methods S1; the complete set of estimates is given in Table S5.

Weights are reported, not just applied. Effective sample size and the distribution of weights are given for each arm, and Figure 2 shows the propensity overlap alongside them. An E-value was calculated for the weighted risk ratio to express how strong an unmeasured confounder would have to be to explain the result [26].

A sensitivity analysis assessed the potential impact of unrecorded events. Because a record-based follow-up can still miss an event, we asked how much unrecorded outcome the finding could absorb. Unobserved events were added to the guidewire-first arm until the weighted difference reached zero. They were added first to the patients whose weights erase it fastest and then to those whose weights erase it slowest, so that the answer is bounded from both sides. The propensity model was not refitted because it uses covariates only and an unobserved outcome does not move a weight.

Checks on covariate linearity, propensity-model size and collinearity, and three further interval estimators for the small number of clusters, a patient-level bootstrap, a wild cluster bootstrap and a cluster-robust CR2 (bias-reduced linearisation) interval, are described in the Supplementary Methods S1.

Components of the exploratory any-grade composite are reported as weighted risk differences with intervals, as exploratory effects rather than tests. No component *p* value is reported, and no multiplicity correction is applied to them, because a corrected *p* value would belong to an unweighted test that appears in no table and no figure. Markers of guidewire failure are reported unadjusted. The arm holds 106 men and 16 failures, so a multivariable model would give unstable estimates, and none was fitted; these comparisons are hypothesis-generating.

Balance is reported as standardised differences rather than *p* values, for the reason given in the footnote to Table 1. Two subgroups were examined. The cohort was split by active antithrombotic use and, separately, by whether the urethra was already bleeding at time zero; an unweighted risk difference was estimated within each stratum, and for the antithrombotic split the difference between strata is reported with a Wald interval. Neither stratum is large enough to support a propensity model of its own, so the stratum estimates are not weighted. Both splits were chosen because bleeding risk is the clinical reason a urologist hesitates to puncture; neither was selected after inspecting outcomes, and both are reported as exploratory.

Analyses were performed in Python 3.14.3 with numpy 2.4.4, pandas 2.3.3, scipy 1.17.1, statsmodels 0.14.6 and matplotlib 3.10.9. A large language model (Claude Opus 5, Anthropic; accessed through a command-line interface in August 2026) was used to write analysis, table and figure code and to draft and revise manuscript prose. No patient-level data were supplied to the model: code was executed locally against the dataset, and every reported value is an output of that code rather than of a language model. The authors verified the code, outputs and references and are responsible for the content. Every quantity computed from these data was produced by a single specification file in one pass; the screening count and the figures quoted from published studies are attributed where they appear. Derived variables, covariate sets and estimators are specified in full above and in Supplementary Methods S1.

### 2.6. Ethics

The study was conducted in accordance with the Declaration of Helsinki and approved by the Scientific Research Ethics Committee of the Republic of Türkiye Ministry of Health, University of Health Sciences, Ümraniye Training and Research Hospital, Istanbul, Türkiye (protocol code 202, approved 13 May 2026). The requirement for informed consent was waived given the retrospective design and the use of routinely collected data. The study is reported according to the Strengthening the Reporting of Observational Studies in Epidemiology (STROBE) statement for cohort studies.

## 3. Results

### 3.1. Cohort and Treatment Assignment

The cohort was assembled over seven years. In all, 352 men met the entry definition between January 2019 and December 2025. Of these, 246 went directly to suprapubic cystostomy and 106 to a guidewire attempt. Figure 1 traces the cohort from presentation to the drainage each man left with: 262 with a suprapubic catheter and 90 with a urethral one.

Table 1 sets out the two arms at time zero. The differences follow bedside logic rather than any protocol: oral anticoagulants were three times more common among the men taken to a guidewire attempt (22.6% against 6.9%). Known urethral stricture ran the other way. The propensity model had a c-statistic of 0.697, and propensity scores spanned 0.06 to 0.88. Overlap weighting gives least weight to men whose scores fall outside the range observed in the other arm: 3 in the guidewire arm and 14 in the direct arm, carrying 1.3% of the total weight between them.

### 3.2. Guidewire Success

Guidewire-assisted catheterisation was successful in most men. It placed a urethral catheter in 84.9% (90/106; 95% CI 76.9 to 90.5) of those in whom it was tried, and the remaining 16 crossed over to cystostomy while remaining in their assigned arm. At the route-specific event rate observed among the men who received a cystostomy, those 16 crossovers would be expected to contribute about one such event, so the design exposes the guidewire-first arm to cystostomy harm, but the cohort is too small to demonstrate it. Median recorded total procedure duration was 12.7 min (IQR 10.4–15.0) in the direct-cystostomy arm and 14.0 min (IQR 11.3–19.4) in the guidewire-first arm; it was 18.2 min (IQR 14.0–21.5) among the 16 guidewire attempts that failed. These durations begin with the rescue procedure and do not measure time from presentation to definitive drainage.

### 3.3. Primary and Secondary Outcomes

Overall strategy-associated complications were less frequent after a guidewire attempt. A new Clavien–Dindo grade II or higher event within 30 days affected 14.6% (36/246) of the direct-cystostomy arm and 6.6% (7/106) of the guidewire-first arm. After overlap weighting, the risks were 17.6% and 5.3%, a weighted absolute difference of −12.2 (−19.0 to −5.5) percentage points, with intervals resampling the fifteen operators. Counting every recorded grade II or higher event instead, prevalent infection included, yielded −13.3 (−22.7 to −3.1). A Clavien–Dindo grade of III or higher occurred in 7.7% (19/246) of the direct arm and 0.0% (0/106; 95% CI 0.0 to 3.5) of the guidewire-first arm. No patient in either arm died within 30 days, so no outcome was truncated by a competing risk.

Adjustment enlarged the difference rather than shrinking it. The largest imbalance ran against the guidewire arm. Anticoagulated men were concentrated there, and the direction of that imbalance could have attenuated rather than created the association, so the unweighted comparison may have understated the contrast. The same imbalance gives the weighting its leverage: 17 anticoagulated men in the cystostomy arm carry 8 of its events, and a propensity model refitted after excluding anticoagulated men altogether gives −8.9 (−15.7 to −2.1) percentage points. The weighted estimate therefore leans on a small, event-dense cell. Weighting equalised the weighted mean of every covariate entered in the propensity model, the largest standardised difference among them falling from 0.453 to zero; variables outside the model are not balanced by it. Effective sample size was 97 in the guidewire-first arm and 200 in the cystostomy arm. The raw overlap weights had a median of 0.31, a 90th percentile of 0.72 and a maximum of 0.89; expressed as a share of the summed weight, no single patient carried more than 0.7%; the pooled spread is bimodal by construction, the weight being the propensity score in one arm and its complement in the other; Figure 2 shows the propensity overlap and the weight distribution.

**Figure 3:**
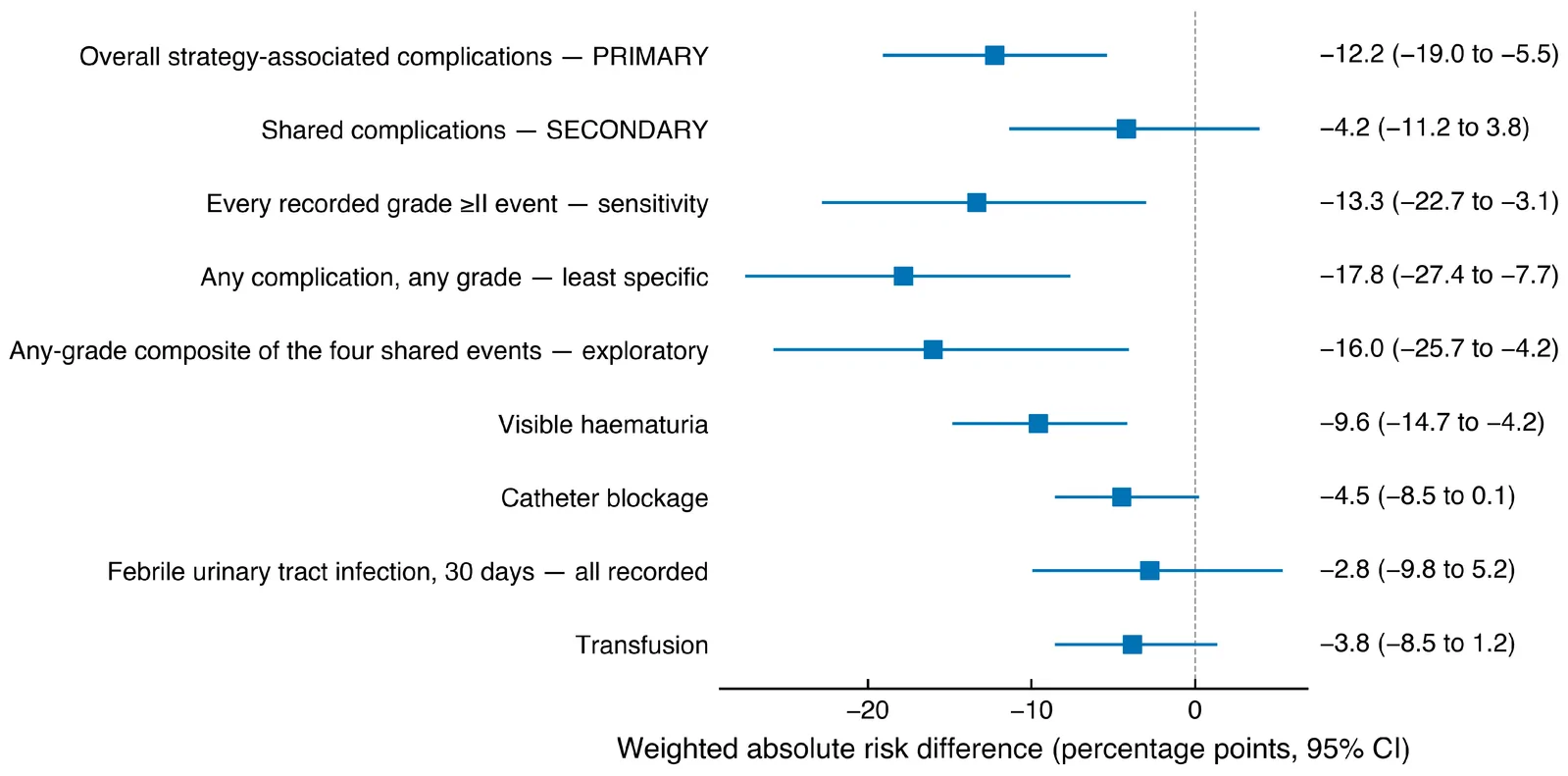
Overlap-weighted absolute risk differences, guidewire-first minus direct cystostomy, in percentage points with 95% confidence intervals resampled over operators. The rows follow the outcome hierarchy: the primary outcome, overall strategy-associated complications, any new Clavien–Dindo grade II or above complication within 30 days; then shared complications, the secondary outcome that removes route-specific events at the same grade threshold; then two sensitivity rows, the composite counting every recorded grade II or above event and any complication of any grade; then the exploratory any-grade composite of the four shared events and its four components. All estimates are overlap-weighted. The scale is linear and referenced to zero because the estimand is a difference rather than a ratio. The dashed vertical line marks a difference of zero.

The device events carried much of that difference. Shared complications, at the same grade threshold but with route-specific events removed, gave a smaller estimate that no longer excluded zero: 6.9% (17/246) against 6.6% (7/106) unweighted, and −4.2 (−11.2 to 3.8) percentage points after weighting. Much of the primary difference therefore rests on the events only a cystostomy can produce. An exploratory any-grade composite of the four shared events differed by −16.0 (−25.7 to −4.2) percentage points, but it lowers the grade threshold, removes route-specific events and counts infection present at time zero, so it changes three things at once and cannot separate them. Component estimates appear in Figure 3 and Table 2. Every one of the 13 transfusions, 10 in the cystostomy arm and 3 in the guidewire-first arm, occurred in a man with visible haematuria recorded.

### 3.4. Sensitivity Analyses

Neither the operator nor the weighting scheme carried the result; Table 2 gives the principal specifications and Table S5 every estimator. A propensity model containing operator fixed effects, which treats the operator as a possible confounder and not only as a cluster, gave −14.1 (−20.2 to −7.2) percentage points against −12.2 (−19.0 to −5.5) for the primary analysis. Stabilised inverse-probability weights gave −11.7 (−17.1 to −4.1) and a doubly robust estimator −10.2 (−16.1 to −1.3); both target the whole cohort rather than the overlap population and are therefore close but not identical estimands. Adding year of procedure gave −12.2 (−18.9 to −5.4). Omitting each operator in turn moved the estimate from −14.0 to −10.3 percentage points, and 0 of 2000 bootstrap resamples failed to yield an estimate.

Two small-cluster corrections agreed with the primary interval. A wild cluster bootstrap gave −12.2 (−19.7 to −4.8) percentage points (*p* = 0.006), and a cluster-robust CR2 interval on 13.2 degrees of freedom gave −12.2 (−19.5 to −5.0). Neither crossed zero. Both are slightly wider than the primary interval, which is the direction a percentile cluster bootstrap is expected to err in with fifteen clusters; both hold the propensity weights fixed, so they account for the clustering and not for the estimation of the weights.

The same sensitivity suite was run for shared complications and is reported in Table S5. Stabilised inverse-probability weights gave −3.9 (−10.0 to 5.4) percentage points, a doubly robust estimator −2.1 (−8.4 to 9.1), a propensity model containing year of procedure −4.1 (−10.9 to 3.9), one containing operator fixed effects −6.5 (−13.4 to 2.5), the clinically restricted model −3.3 (−10.2 to 4.4), a patient-level bootstrap −4.2 (−10.3 to 1.6), a wild cluster bootstrap −4.2 (−12.5 to 4.1) (*p* = 0.32) and a cluster-robust CR2 interval −4.2 (−12.4 to 4.0). Every one of them includes no difference. For the primary outcome, 10 to 23 unrecorded grade II or higher events among the 99 event-free guidewire-first men (10% to 23%) would move the weighted difference to zero. For shared complications, the tipping point is correspondingly closer: 4 to 12 unrecorded events among the 99 event-free guidewire-first men (4% to 12%) would move it to zero.

Neither model size, resampling unit, outcome definition, nor bleeding status at time zero moved the primary estimate. The five clinically chosen covariates gave −11.9 (−17.8 to −5.3) percentage points, and resampling patients rather than operators gave −12.2 (−19.2 to −5.7). Counting every recorded grade II or higher event, prevalent infection included, gave −12.1 (−19.7 to −4.5) unweighted and −13.3 (−22.7 to −3.1) weighted; that composite carries 20 men whose only qualifying event was an infection they already had. Among the men whose urethra was not already bleeding at time zero, the unweighted difference was −9.3 (−17.4 to −1.2) percentage points; among those already bleeding, it was −6.0 (−16.7 to 4.8).

The direction held in both antithrombotic strata. Only one interval excluded no difference, and these stratum estimates are unweighted because neither stratum is large enough to support a propensity model of its own. Men on an antithrombotic agent showed −12.9 (−22.8 to −3.0) percentage points and men on none −4.5 (−12.6 to 3.7). The larger estimate fell in the stratum with the higher bleeding risk, but the two strata did not separate. The difference between them was −8.4 (−21.2 to 4.4) percentage points (*p* = 0.20). They are too small to establish that the strategies differ by antithrombotic status.

The weighted risk ratio was 3.29 (95% CI 1.56 to 13.82), its interval from the same operator-level bootstrap as the primary analysis. Its E-value, computed for direct cystostomy versus guidewire-first, was 6.04 at the point estimate and 2.49 at the lower confidence limit. The second bounds how weak an unmeasured confounder could be and still move the result to the null.

### 3.5. Route-Specific Events

Each route produced its own harms. Table 3 reports the suprapubic events without comparison and the urethral event for both arms. Among the 262 men who received a cystostomy, malposition occurred in 4.6% (12/262), loss of the tract in 2.3% (6/262) and bowel injury in 0.4% (1/262). The bowel injury occurred in an obese man with previous lower abdominal surgery; it declared itself on the first day with abdominal pain, guarding and rebound tenderness, and was found at laparotomy on the second. Reintervention within 30 days was needed in 6.9% (18/262), and every one of those procedures addressed a malposition or a lost tract.

Recorded urethral events were infrequent. Among the men whose urethra was not bleeding when the rescue began, new meatal bleeding appeared in 1.4% (1/74; 95% CI 0.2 to 7.3) of the guidewire-first arm and 0.0% (0/156; 95% CI 0.0 to 2.4) of the direct arm, a difference of +1.4 (−1.3 to 4.0) percentage points. The single event was Clavien–Dindo grade I. Both arms were checked against the file by the same criterion, so this comparison rests on a symmetric ascertainment.

A failed attempt did not appear to compromise the cystostomy that followed. None of the 16 crossover patients had a malposition, a lost tract or a reintervention, although that number of patients cannot exclude a rate as high as roughly one in five.

### 3.6. Markers of Guidewire Failure

Several baseline characteristics were associated with failure in exploratory analyses. Within the guidewire-first arm, the attempt failed in 16 of 106 men. Four markers separated those men from the rest. The attempt failed in 4 of 6 men with a suspected bladder-neck contracture against 12 of 100 without; in 7 of 14 with a known urethral stricture against 9 of 92 without; in 6 of 14 with a prior transurethral or laser prostatectomy against 10 of 92 without; and in 8 of 21 with a previously difficult catheterisation against 8 of 85 without. Prior urethral surgery resulted in an interval that included no difference. Table S6 gives the odds ratio, the Fisher exact *p* value and the raw failure counts for each. With 16 failures, these comparisons are unadjusted and hypothesis-generating.

## 4. Discussion

Ninety of the 106 wire attempts avoided a suprapubic tube. The guidewire-first strategy was associated with fewer overall strategy-associated complications than proceeding directly to cystostomy. The weighted absolute difference was −12.2 (−19.0 to −5.5) percentage points, and −13.3 (−22.7 to −3.1) when every recorded grade II or above event was counted instead. For shared complications, at the same grade threshold but with route-specific events removed, the difference was −4.2 (−11.2 to 3.8) points, an interval that includes no difference. The observed contrast therefore rests largely on events that only a cystostomy can produce. For the clinician called to a failed catheterisation, the finding bears on the first move rather than on eventual drainage; the failure markers describe whom to prepare for rather than whom to exclude.

### 4.1. What the Finding Answers at the Bedside

The question a urologist faces is not whether the wire works. It is what an attempt costs when it fails. Two costs are usually feared: that the attempt injures the urethra, and that the delay and instrumentation make the subsequent cystostomy worse. Delay to definitive drainage was never recorded and could not be assessed; the recorded urethral harm is set out below.

Recorded urethral harm after the attempt was minimal. New meatal bleeding after the attempt occurred in 1.4% (1/74; 95% CI 0.2 to 7.3) of the men whose urethra was not already bleeding, against 0.0% (0/156; 95% CI 0.0 to 2.4) in the direct arm. The event was Clavien–Dindo grade I. None of the 16 men who crossed over had a malposition, a lost tract or a reintervention.

Both figures rest on small denominators. Sixteen crossover patients cannot exclude a complication rate as high as roughly one in five. A single event among the 74 men whose urethra was not already bleeding leaves the upper confidence limit for new bleeding at seven per cent. The claim the data support is narrow: no harm was recorded at a magnitude this cohort could detect. Surveillance for meatal bleeding was symmetric, but the set of urethral harms a record can hold is not, as Section 4.3 sets out.

The cost of a cystostomy is not the same for every man. Among those who received one, a route-specific event occurred in 2.9% (2/70) in the leanest body-mass index stratum against 23.1% (3/13) in the heaviest, rising across the four strata set out in Table 3. The numbers in the upper strata are small, and no comparison is made, but the gradient runs in the way a trocar puncture through a thick abdominal wall would predict, and the single bowel injury occurred in an obese man with previous lower abdominal surgery. The wire did not become less reliable in the same men: it placed a catheter in 85.7% (24/28) of the leanest stratum, 82.5% (33/40) of the second leanest and 86.1% (31/36) of the second heaviest. Only two men in the heaviest stratum underwent a guidewire attempt at all, so that stratum carries no usable estimate. If a guidewire attempt has any benefit, it is greatest where the puncture is hardest, and this cohort cannot test that in the heaviest stratum.

Several baseline characteristics were associated with failure. A suspected bladder-neck contracture, a known stricture, a prior prostatectomy or a previously difficult catheterisation each accompanied a higher failure rate in exploratory comparisons on 16 events. None of this amounts to a triage rule. The attempt still succeeded more often than it failed in men with a prior difficult catheterisation, prior urethral surgery or previous prostate resection. Failure was at least as common in the other two, a suspected bladder-neck contracture and a known stricture, but those rest on six and fourteen men. They describe whom to prepare for, not whom to skip.

### 4.2. Relation to Previous Work

Reported success rates for wire-assisted catheterisation sit above ours. A prospective multicentre series succeeded in 107 of 115 attempts, but the operators were doctors without urology training using a Seldinger technique [13]. A purpose-built catheterisation device with an integrated wire has been evaluated in two separate cohorts [14,15]; no such device was available here, so the wire was passed through an opening made in a standard catheter. Where blind or cystoscopy-assisted catheterisation has already failed, fluoroscopic placement by interventional radiologists succeeds again [17,18]. Our figure comes from urologists at the bedside with a wire and an ultrasound probe, and the difference between these settings is resource, not technique alone.

The suprapubic side has better benchmarks than the urethral one. The meta-analysis published with the national suprapubic-catheter audit cited in the Introduction reported bowel injury at 0.7%, and the audit advised counselling low-risk patients with a figure below 0.25% [7]. A series of 1000 elective procedures at a spinal injuries centre produced a Clavien–Dindo grade of IIIb or higher in 0.6% of procedures [27]. Our single bowel injury in 262 cystostomies sits between that counselling figure and the pooled estimate, and a scoping review of the same complication describes management but finds no consensus [28]. The other rates do not compare so simply: that series was elective, performed largely in patients with a spinal cord injury, and used cystoscopic or Lowsley-guided placement rather than the single-puncture ultrasound-guided technique used here in an emergency.

The comparative trials cited in the Introduction answer a different question [19]. Those trials randomised patients in whom either route was available. In the population studied here, the urethral route has already failed, which is what makes the choice a rescue decision rather than a preference.

The clinical importance of that decision is established. In one prospective series, traumatic catheterisation occurred in 6.7 per 1000 catheters inserted, and 24% of the injured required an indwelling suprapubic catheter [4]. Later series and long-term follow-up describe the same injuries and their sequelae [5,6,29]. A suprapubic tube is therefore already a common endpoint of a catheterisation injury, and the question is how often that endpoint can be avoided and at what cost.

### 4.3. Two Methodological Points

The comparison is built at strategy level, not device level, and it evaluates initial rescue strategies rather than the intrinsic safety of either procedure. Every complication counts under the arm a man was assigned to. That includes the events only one route can produce, because a cystostomy that is never placed is a consequence of the strategy rather than an artefact of how the arms were named. Crossover keeps the guidewire-first arm exposed to cystostomy complications, so the contrast is not fixed by the design, although no malposition, lost tract or reintervention occurred among the men who crossed over. The asymmetry that remains is one of measurement rather than of naming. Every route-specific event that could reach grade II in this record belongs to the suprapubic route: a malposition, a lost tract or a bowel injury. The urethral route has no counterpart at that grade. New meatal bleeding was assessed in both arms by the same criterion and graded I, so it cannot meet the grade-II threshold; stricture falls outside a 30-day window. A false passage, a balloon inflated in the urethra or an early stricture would all have been invisible here. The primary outcome therefore counts the device harms of one route and cannot count those of the other, and the shared-complications outcome is the one that compares like with like: −4.2 (−11.2 to 3.8) weighted, −0.3 (−6.0 to 5.4) unweighted. That outcome was put through the same sensitivity suite as the primary one, and no estimator moved it away from no difference; what the interval leaves open is a benefit of up to eleven percentage points or a harm of up to four, so the finding is compatibility with no difference rather than evidence of none. Pairing the two outcomes in this way may be worth applying wherever two procedures end in different hardware, though this single centre cannot test that. Estimate at the level of the strategy, then hold the severity threshold fixed and remove the device events to see what remains. Changing the event set and the threshold together cannot separate the two.

The second point concerns a measurement rather than an analysis. A field labelled iatrogenic urethral trauma in fact recorded blood at the meatus rather than an injury, and it was withdrawn for that reason. A separate field records the sign itself, and it shows why the label mattered: the blood was documented in the file as already present before the rescue in 122 of the 123 men who had it, and it persisted unchanged through a procedure that never touched the urethra. Coding meatal blood as a procedural complication attributes to the rescue what the attempts before it produced. Guidance treats the sign the same way: the European Association of Urology lists blood at the meatus as the cardinal sign of urethral injury while noting that its absence does not exclude one [9]. A sign that neither confirms nor excludes an injury cannot define the outcome of a comparison.

### 4.4. Limitations

Selection is the main threat here. This was a single-centre retrospective cohort, and the strategy was chosen by the treating urologist. The propensity model showed modest discrimination (c-statistic 0.697). Discrimination measures how well the covariates separate the two arms, not how much of the choice they explain, so it neither rules unmeasured selection out nor sizes it; no measure that would is available here. Clinically important unmeasured selection therefore remains possible.

An E-value bounds one mechanism only. The E-value of 6.04 for the weighted risk ratio describes how strong a single hidden confounder would have to be to account for the point estimate, and 2.49 how strong it would have to be to move the lower confidence limit to the null. Fifteen urologists chose the strategy without a protocol, and anatomical difficulty at the bedside, operator competence and the number and character of the preceding attempts are not in the record. The last of these is likely the largest source of residual confounding: a man referred after one gentle failed attempt differs from one referred after several traumatic ones, both in the strategy he is offered and in his risk of complication. Frailty is a further unmeasured candidate in a cohort of this age, since it plausibly influences both the strategy chosen and the risk of complication [30]. The E-value speaks to none of that: it addresses a single unmeasured confounder acting on both arms alike, and says nothing about differential ascertainment, outcome misclassification, or a selection mechanism that no one covariate represents.

The anticoagulant imbalance may run the other way. Anticoagulated men were concentrated in the guidewire arm, which is what a urologist reluctant to puncture an anticoagulated abdomen would produce, and that imbalance could have attenuated rather than created the difference. It cuts both ways: those men are also where the weighting draws its leverage, and excluding them lowers the estimate to −8.9 (−15.7 to −2.1) percentage points.

Ascertainment is the second threat, though narrower than a single-centre record would make it. Every patient was followed in the national personal health record as well as this hospital, so care taken at another reporting institution was visible. What no record shows is an event that produced no reported encounter. Whether that residue falls unequally between the arms cannot be determined here.

An unequal residue would change what the estimate means. If events are captured more completely after a cystostomy, part of the difference reported here is a difference in counting rather than in risk. The residue is narrow. Because follow-up used the national record as well as this hospital’s, an event managed anywhere in the health system, primary care included, is visible; what remains outside is an event resolved with no recorded contact at all. The record does not mark whether an event occurred before or after discharge, so events cannot be split by that boundary and the share exposed to this asymmetry cannot be quantified.

Under-ascertainment would have to be large. The amount required to erase the estimate is substantial relative to the 99 guidewire-first men without a recorded event: 10 to 23 additional grade-II-or-higher events (10% to 23%), depending on which weighted patients they affected. The records cannot determine whether a shortfall of that size in one arm is plausible, and the analysis does not exclude it.

One source of misclassification was removed by definition. Counting an infection only in men who were not already infected at time zero excludes 20 men whose only qualifying event was a condition they arrived with, every one of them grade II. Keeping those men, as an audit of the record alone would, moves the weighted estimate to −13.3 (−22.7 to −3.1) and the unweighted contrast to −12.1 (−19.7 to −4.5). The weighted estimate moves less because infection at presentation is one of the covariates the weights balance. That disposes of one source of misclassification. It does not bound differential loss to follow-up, which remains unmeasured.

The exploratory any-grade composite is carried by its milder components. Visible haematuria carries most of it: −9.6 (−14.7 to −4.2) percentage points, the only component whose interval excludes no difference. The other three are smaller, and their intervals overlap each other, so they cannot be ranked against one another here. Grade III or higher events were confined to the cystostomy arm. That establishes the severity of what a cystostomy costs when it is used; it is not independent evidence that the contrast is robust, for the reason given in Section 4.3. The wider composite was not specified in advance and is reported as a sensitivity analysis; the shared-complications outcome was defined to answer the objection that the contrast rests on route-specific events, rather than being fixed beforehand. Table 2 reports every component for that reason.

One number is missing from the record. The interval from presentation to definitive drainage was not recorded, so the delay that follows a failed guidewire attempt could not be measured. The recorded procedure durations reported in Section 3.2 begin with the rescue procedure and do not capture that wait. The guidewire-first strategy therefore carries an unmeasured cost for the 16 men of 106 in whom the wire failed; in acute retention, that delay may be clinically important. A second cost is not measured either. The 90 men who left with a urethral catheter did so through a urethra that had already defeated one attempt, and the record holds no field for what happened at the first catheter change or trial of void.

Three remaining limits are important. A 30-day window is too early for stricture formation, the outcome that matters most after urethral instrumentation [5]. Outcome abstraction and the pre-analysis corrections were performed by a single reviewer. Although a duplicate-adjudication sample was prepared, it was not completed by a second reviewer; no inter-rater agreement estimate is therefore available. Interval estimation at the operator level also remains uncertain with only fifteen clusters, which the patient-level bootstrap, the leave-one-operator-out refits and the wild cluster and CR2 intervals bound but do not remove. A prospective comparison should record time to definitive drainage, patient discomfort, the need for subsequent urethral procedures, urethral outcomes beyond 30 days, and a validated frailty measure.

The result applies to a narrow setting. It was produced by urologists at one tertiary centre, in men, using a hydrophilic guidewire whose position was confirmed on ultrasound and a single-puncture trocar cystostomy. It should not be read across to women, to children, to pelvic trauma or a known complete urethral disruption, or to centres where ultrasound is not at the bedside.

Markers of guidewire failure rest on 16 events. A multivariable model would be unstable at that number, and none was fitted; the odds ratios are unadjusted, and their intervals are wide. They are reported to describe this cohort, not to support a triage instrument.

## 5. Conclusions

Among men assigned to guidewire-first, 84.9% avoided a suprapubic tube. After a failed urethral catheterisation, a guidewire attempt was associated with fewer overall strategy-associated complications recorded within 30 days than proceeding directly to cystostomy, a difference of −12.2 percentage points, among men whose baseline profiles appeared under both strategies. For shared complications that either route could produce, the difference was −4.2 percentage points and compatible with none. The characteristics that accompanied a failed attempt indicate whom to prepare for, not whom to exclude. These findings support prospective evaluation of a guidewire-first strategy and may inform the rescue decision when both strategies are feasible; they do not establish that the wire is safer.

## Figures and Tables

**Table 1:** Baseline characteristics by initial rescue strategy.

Characteristic	Direct Suprapubic Cystostomy (*n* = 246)	Guidewire-First (*n* = 106)	Signed SMD
***Demographic and clinical***			
Age, years	69.0 (IQR 63.0–76.0)	69.0 (IQR 62.0–77.8)	0.089
Body-mass index, kg/m^2^	27.45 (IQR 24.80–30.60)	28.10 (IQR 24.78–31.40)	0.077
Retention volume, mL	865.5 (IQR 708.3–1026.0)	867.0 (IQR 694.5–1102.5)	0.185
Haemoglobin, g/dL	13.30 (IQR 11.93–14.30)	12.85 (IQR 11.70–14.50)	−0.050
Platelets, ×10^9^/L	236.5 (IQR 187.0–281.5)	243.5 (IQR 188.8–287.8)	0.104
INR	1.00 (IQR 0.92–1.08)	1.00 (IQR 0.91–1.12)	0.411
Creatinine, mg/dL	1.19 (IQR 0.95–2.39)	1.14 (IQR 0.90–2.42)	0.004
***Lower urinary tract history***			
Known benign prostatic hyperplasia	67.9% (167/246)	74.5% (79/106)	0.147
Known urethral stricture	21.5% (53/246)	13.2% (14/106)	−0.221
Suspected bladder-neck contracture	10.2% (25/246)	5.7% (6/106)	−0.167
Prior TUR-P or HoLEP	18.3% (45/246)	13.2% (14/106)	−0.139
Prior urethral surgery	10.6% (26/246)	10.4% (11/106)	−0.006
Prior difficult catheterisation	23.2% (57/246)	19.8% (21/106)	−0.082
Prior lower abdominal surgery	17.9% (44/246)	17.0% (18/106)	−0.024
***Comorbidity and haemostasis***			
Diabetes mellitus	26.4% (65/246)	27.4% (29/106)	0.021
Coronary artery disease ^a^	26.0% (64/246)	26.4% (28/106)	0.009
Chronic kidney disease ^a^	12.6% (31/246)	12.3% (13/106)	−0.010
Urinary tract infection at presentation	30.5% (75/246)	22.6% (24/106)	−0.178
Urethral bleeding before rescue	36.6% (90/246)	30.2% (32/106)	−0.136
Any antithrombotic agent ^a^	47.2% (116/246)	60.4% (64/106)	0.267
Oral anticoagulant	6.9% (17/246)	22.6% (24/106)	0.453
Antiplatelet agent	40.2% (99/246)	37.7% (40/106)	−0.051

SMD, standardised mean difference, signed as guidewire-first minus direct suprapubic cystostomy; a negative value indicates the characteristic was more common in the direct arm. ^a^ Not entered in the propensity model. Table S3 reports the balance of these three variables before and after weighting. Continuous variables are median (interquartile range); binary variables are % (*n*/N), with 95% confidence intervals omitted for brevity. No value was missing for any variable shown. Standardised differences are reported in place of *p* values. A *p* value here measures detectability, not imbalance: it confounds the size of a difference with the size of the arm, and the guidewire-first arm holds 106 patients. Of the 19 covariates in the propensity model, 10 exceed a standardised difference of 0.10 while 1 reaches *p* < 0.05 by Fisher exact test for binary variables and the Mann–Whitney test for continuous ones; a *t* test on the means would return a different count, which is itself the reason standardised differences are preferred here. Standardised differences are given for every baseline variable; those not entered in the propensity model are balanced only to the extent that they correlate with variables that were. TUR-P, transurethral resection of the prostate; HoLEP, holmium laser enucleation of the prostate; INR, international normalised ratio. Medians can coincide while the standardised difference is large, because the standardised difference is computed from means and standard deviations: the INR rows share a median of 1.00 but differ in the upper tail, which is what the difference of 0.411 reflects. IQR, interquartile range; INR, international normalised ratio; TUR-P, transurethral resection of the prostate; HoLEP, holmium laser enucleation of the prostate.

**Table 2:** Overall strategy-associated complications, shared complications, principal sensitivity analyses, wider outcome definitions and exploratory shared-event composite, by initial strategy.

Outcome	Direct Suprapubic Cystostomy	Guidewire-First	Absolute Risk Difference, Percentage Points (95% CI)
***Overall strategy-associated complications (primary outcome): new grade ≥II event, either route***			
New Clavien–Dindo grade ≥II, 30 days—unweighted	14.6% (36/246)	6.6% (7/106)	−8.0 (−14.5 to −1.6)
New Clavien–Dindo grade ≥II, 30 days—overlap-weighted	17.6%	5.3%	−12.2 (−19.0 to −5.5)
***Shared complications (secondary outcome): same grade threshold, route-specific events removed***			
New grade ≥II with a shared event—unweighted	6.9% (17/246)	6.6% (7/106)	−0.3 (−6.0 to 5.4)
New grade ≥II with a shared event—overlap-weighted	9.5%	5.3%	−4.2 (−11.2 to 3.8)
***Principal sensitivity analyses of the primary outcome***			
Propensity model including operator fixed effects	18.2%	4.2%	−14.1 (−20.2 to −7.2)
Doubly robust estimator	15.1%	4.9%	−10.2 (−16.1 to −1.3)
Stabilised inverse-probability weights	16.6%	4.9%	−11.7 (−17.1 to −4.1)
Clinically restricted propensity model (5 covariates)	17.7%	5.9%	−11.9 (−17.8 to −5.3)
***Sensitivity analyses: wider outcome definitions***			
Every recorded grade ≥II event, prevalent infection included	22.3%	8.9%	−13.3 (−22.7 to −3.1)
Any complication, any grade	31.7%	13.9%	−17.8 (−27.4 to −7.7)
***Exploratory any-grade composite of the four shared events***			
Composite—overlap-weighted	24.9%	8.9%	−16.0 (−25.7 to −4.2)
Visible haematuria	12.0%	2.4%	−9.6 (−14.7 to −4.2)
Catheter blockage	5.5%	1.0%	−4.5 (−8.5 to 0.1)
Febrile urinary tract infection, 30 days—all recorded	9.3%	6.6%	−2.8 (−9.8 to 5.2)
Transfusion	6.2%	2.4%	−3.8 (−8.5 to 1.2)

Values are % (*n*/N) for unweighted rows and weighted or standardised percentages for the other rows. Unweighted risk-difference intervals use the normal approximation; all other intervals use the operator-level bootstrap. Doubly robust percentages are standardised over the whole cohort by augmented inverse-probability weighting; stabilised inverse-probability and doubly robust rows therefore target the whole cohort rather than the overlap population. Risk differences were computed from unrounded risks and may differ in the last decimal place from the difference in the rounded percentages. The clinically restricted model includes oral anticoagulant use, infection at presentation, an anatomical obstruction, a prior transurethral or laser prostatectomy, and age. The doubly robust outcome model estimates 21 parameters from 43 events, 2.0 per parameter; its estimate is read for the direction of the difference, not its size. The primary outcome counts every complication arising after time zero under the strategy first attempted, including events only one route can produce; crossover keeps the guidewire-first arm exposed to cystostomy complications. A condition present at time zero does not count, which affects febrile urinary tract infection alone. The shared-complications outcome keeps the grade II bar and requires at least one event either strategy could produce. The exploratory composite additionally drops the grade requirement and counts febrile infection present at time zero, so it changes three things at once; it cannot separate them. CI, confidence interval. The four component rows count every recorded event of that type, including a febrile infection present at time zero; they describe the exploratory any-grade composite and not the primary outcome. The any-grade row counts every complication recorded in the file, including 5 grade I entries whose only record is the iatrogenic-urethral-trauma field, withdrawn from every other outcome because it recorded meatal blood rather than injury (Section 4.3); all of them fall in the guidewire-first arm. It is the least specific row in the table. Raw counts for the four components, direct cystostomy against guidewire-first: visible haematuria 10.2% (25/246) and 2.8% (3/106); catheter blockage 6.1% (15/246) and 0.9% (1/106); febrile infection 11.0% (27/246) and 6.6% (7/106); transfusion 4.1% (10/246) and 2.8% (3/106). Estimates for both outcomes under every alternative estimator and propensity specification, including the interval estimators for fifteen clusters, are given in Table S5; specifications are in the Supplementary Methods S1.

**Table 3:** Route-specific adverse events and overall severity.

Event or Stratum	Denominator	Events, % (*n*/N)
***Suprapubic route***		
Cystostomy malposition	Received a cystostomy	4.6% (12/262)
Loss of the cystostomy tract	Received a cystostomy	2.3% (6/262)
Bowel injury	Received a cystostomy	0.4% (1/262)
Reintervention under local anaesthesia within 30 days	Received a cystostomy	6.9% (18/262)
***Urethral route***		
New meatal bleeding, guidewire-first	Urethra not bleeding before rescue	1.4% (1/74; 95% CI 0.2 to 7.3)
New meatal bleeding, direct cystostomy	Urethra not bleeding before rescue	0.0% (0/156; 95% CI 0.0 to 2.4)
***Route-specific events by body-mass index, kg/m^2^***		
≤25	Received a cystostomy	2.9% (2/70)
>25 to 30	Received a cystostomy	3.4% (4/117)
>30 to 35	Received a cystostomy	16.1% (10/62)
>35	Received a cystostomy	23.1% (3/13)
***Overall severity***		
Clavien–Dindo grade III or higher, direct cystostomy	All patients in the arm	7.7% (19/246)
Clavien–Dindo grade III or higher, guidewire-first	All patients in the arm	0.0% (0/106; 95% CI 0.0 to 3.5)

Events in the suprapubic block can arise under one route only and are reported as description, by the route that produces them. New meatal bleeding was assessed in both arms by the same criterion and is therefore shown for each. The suprapubic events are not excluded from the primary outcome, which counts every complication under the strategy first attempted. Rates in the suprapubic and urethral blocks are as-treated, by the route the patient received. The overall-severity rows at the foot of the table are not route-specific: they are by initial strategy and are shown to place the route-specific events on the grade scale rather than to compare the arms. New meatal bleeding is defined only in patients whose urethra was not already bleeding when the rescue began. Rates by body-mass index are descriptive and are shown to indicate in whom a cystostomy is most costly; no comparison between arms is implied, and no test is reported. The laparotomy for the bowel injury is counted in the bowel-injury row and not in the reintervention row, which covers repeat drainage under local anaesthesia.

## Data Availability

Every quantity computed from the study data was produced by a single analysis specification that declares each derived variable, each covariate set and each estimator. That specification and the de-identified patient-level dataset are available from the corresponding author on reasonable request.

## Supplementary Materials

The following supporting information can be downloaded at https://www.mdpi.com/article/10.3390/jcm15207792/s1: Supplementary Methods S1: Sensitivity analyses and model checks, Table S1. Target trial specification and how the observational analysis emulated each component, Table S2. Composition of the primary outcome among the patients who met it: highest Clavien–Dindo grade and event type, by initial strategy, Table S3. Balance diagnostics that overlap weighting does not fix by construction: variance ratios for the continuous covariates in the propensity model, and standardised differences for baseline variables outside it, Table S4. Strategy use by operator, Table S5. Sensitivity analyses of overall strategy-associated complications and of shared complications under alternative estimators and propensity specifications, Table S6. Baseline characteristics associated with guidewire failure, exploratory.

## References

[1] Sliwinski A., D’Arcy F.T., Sultana R., Lawrentschuk N. (2016). Acute urinary retention and the difficult catheterization: Current emergency management. Eur. J. Emerg. Med..

[2] Granieri M.A., Wang H.H.S., Routh J.C., Peterson A.C. (2017). A Nationwide Assessment of the Emergency Department Management of Acute Urinary Retention Due to Urethral Stricture. Urology.

[3] Ha A.S., Pak J., Haas C.R., Miles C., Weiner D.M., Anderson C.B., Badalato G.M. (2021). A Novel Risk Prediction Model to Triage Difficult Urethral Catheterizations. Urology.

[4] Davis N.F., Quinlan M.R., Bhatt N.R., Browne C., MacCraith E., Manecksha R., Walsh M.T., Thornhill J.A., Mulvin D. (2016). Incidence, Cost, Complications and Clinical Outcomes of Iatrogenic Urethral Catheterization Injuries: A Prospective Multi-Institutional Study. J. Urol..

[5] Davis N.F., Bhatt N.R., MacCraith E., Flood H.D., Mooney R., Leonard G., Walsh M.T. (2020). Long-term outcomes of urethral catheterisation injuries: A prospective multi-institutional study. World J. Urol..

[6] Lawton J.D., Ortiz N., Henry A., Smith C., Smith R., Rapp D.E. (2024). Characterization and Outcomes of Iatrogenic Urethral Catheterization Injuries. Urol. Pract..

[7] Hall S., Ahmed S., Reid S., Thiruchelvam N., Sahai A., Hamid R., Harding C., Biers S., Parkinson R. (2019). A national UK audit of suprapubic catheter insertion practice and rate of bowel injury with comparison to a systematic review and meta-analysis of available research. Neurourol. Urodyn..

[8] Jane Hall S., Harrison S., Harding C., Reid S., Parkinson R. (2020). British Association of Urological Surgeons suprapubic catheter practice guidelines-revised. BJU Int..

[9] EAU Guidelines on Urological Trauma (2026). EAU Guidelines, Edn. Presented at the EAU Annual Congress 2026.

[10] Patil B.S., Patil S.B., Kundargi V.S., Patil S.R., Vaidya M.K., Shukla V. (2024). Optimised Protocol for Managing Failed Catheterisation: Leveraging Bedside Retrograde Urethrography and Cystourethroscopy. Cureus.

[11] MacNevin W., Keefe D.T., Milford K., Paterson N.R. (2025). A practical approach to the difficult urethral catheterization for urology trainees. Can. J. Urol..

[12] Lachat M.L., Moehrlen U., Bruetsch H.P., Vogt P.R. (2000). The Seldinger technique for difficult transurethral catheterization: A gentle alternative to suprapubic puncture. Br. J. Surg..

[13] Yuminaga Y., Kam J., Louie-Johnsun M. (2017). Multi-centre, prospective evaluation of the Seldinger technique for difficult male urethral catheter insertions by non-urology trained doctors. BJU Int..

[14] Bugeja S., Mistry K., Yim I.H.W., Tamimi A., Roberts N., Mundy A.R. (2019). A new urethral catheterisation device (UCD) to manage difficult urethral catheterisation. World J. Urol..

[15] Subramanian V., Soni B.M. (2024). Value and Limitations of Urethrotech Catheterisation Device to Manage Difficult Urethral Catheterisation in Male Spinal Cord Injury Patients. Med. Devices.

[16] Vovdenko S., Taratkin M., Morozov A., Yandiev S., Gazimiev M. (2026). A novel device for endoscopy-guided bladder catheterization outside of the operating theatre: Early clinical experience. Int. Urol. Nephrol..

[17] Kim S.W., Nam I.C., Kim D.R., Lee J.S., Kim J.J., Kim B.S., Choi G.M., Park S.E. (2024). Safety and efficacy of fluoroscopy-guided urethral catheterization in case of failed blind or cystoscopy-assisted urethral catheterization. Sci. Rep..

[18] Saiga A., Aramaki T., Sato R., Asahara K. (2024). Fluoroscopy-guided Urethral Catheter Insertion with Guidewire and Catheter for Complex Male Urinary Catheterizations by Interventional Radiologists. Cardiovasc. Interv. Radiol..

[19] Kidd E.A., Stewart F., Kassis N.C., Hom E., Omar M.I. (2015). Urethral (indwelling or intermittent) or suprapubic routes for short-term catheterisation in hospitalised adults. Cochrane Database Syst. Rev..

[20] EAU Guidelines on Urological Infections (2026). EAU Guidelines, Edn. Presented at the EAU Annual Congress 2026.

[21] Hernán M.A., Robins J.M. (2016). Using Big Data to Emulate a Target Trial When a Randomized Trial Is Not Available. Am. J. Epidemiol..

[22] Dindo D., Demartines N., Clavien P.A. (2004). Classification of surgical complications: A new proposal with evaluation in a cohort of 6336 patients and results of a survey. Ann. Surg..

[23] Carson J.L., Stanworth S.J., Guyatt G., Valentine S., Dennis J., Bakhtary S., Cohn C.S., Dubon A., Grossman B.J., Gupta G.K. (2023). Red Blood Cell Transfusion: 2023 AABB International Guidelines. JAMA.

[24] Li F., Morgan K.L., Zaslavsky A.M. (2018). Balancing Covariates via Propensity Score Weighting. J. Am. Stat. Assoc..

[25] Austin P.C. (2009). Balance diagnostics for comparing the distribution of baseline covariates between treatment groups in propensity-score matched samples. Stat. Med..

[26] VanderWeele T.J., Ding P. (2017). Sensitivity Analysis in Observational Research: Introducing the E-Value. Ann. Intern. Med..

[27] Hobbs C., Howles S., Derry F., Reynard J. (2022). Suprapubic catheterisation: A study of 1000 elective procedures. BJU Int..

[28] Jarral F., Hamdy A., Abusand O., Mobayen R., Tokidis E. (2024). Intestinal Injury After Suprapubic Catheterisation: A Scoping Review. Cureus.

[29] O’Connor E.M., Croghan S.M., Baird O., Fallon J., Loughman P., Esoof J., Keenan R.A., Ryan J., Manecksha R., D’Arcy F. (2023). A Prospective Multi-institutional Study Using a Novel Safety Valve for the Prevention of Catheter Balloon Inflation Injury of the Urethra. J. Urol..

[30] Tzelves L., Katsimperis S., Meilak C., Wagg A., Kittang B.R., Baug S., Beisland E.G., Burak Ergül R., Beisland C., Somani B. (2026). Frailty: From concept to clinical practice in urology. Front. Med..

