# Supplementary material for: Guidewire-First Rescue Versus Direct Suprapubic Cystostomy After Failed Urethral Catheterisation: A Propensity-Weighted Cohort Study
Source: J Clin Med. 2026 Oct 9;15(20):7792. doi: 10.3390/jcm15207792 (PMC13705298; doi:10.3390/jcm15207792)
Supplement: Supplementary file 1 [file jcm-15-07792-s001.zip › jcm-4576033-supplementary.pdf]

Supplementary Materials

Guidewire-First Rescue Versus Direct Suprapubic Cystostomy After Failed Urethral Catheterisation: A Propensity-Weighted Cohort Study

Supplementary Methods S1. Sensitivity analyses and model checks

S1.1. Alternative estimators and propensity specifications

Two alternative estimators and one alternative propensity specification were used in place of the primary analysis: stabilised inverse-probability weights instead of overlap weights, a doubly robust estimator, and a propensity model additionally containing calendar year. The doubly robust estimator combined the same propensity model with a logistic outcome model containing the treatment indicator and every covariate as main effects, and was standardised by augmented inverse-probability weighting over the whole cohort. That outcome model is thin: it estimates 21 parameters from 43 events for the primary outcome, 2.0 per parameter, and from 24 events for the shared-complications outcome, 1.1 per parameter. Its estimate is therefore read as a check on the direction of the difference and not on its size.

The intervals for these three estimators, including the doubly robust one, came from the same operator-level bootstrap as the primary analysis. The stabilised inverse-probability and doubly robust estimators reuse the propensity specification of the primary analysis, so they probe the weighting scheme and the outcome model rather than the specification itself. A further specification added operator fixed effects to the propensity model, so that the operator is treated as a possible confounder and not only as a cluster; its estimate is reported in Table 2. That model carries 33 terms; its fitted scores spanned 0.02 to 0.97, the effective sample sizes after weighting were 94 in the guidewire-first arm and 172 in the direct arm, and the largest weight was 0.94.

S1.2. Model size, covariate linearity and collinearity

Two checks addressed the size of the propensity model. Each continuous covariate was expanded as a natural cubic spline with 3 knots, placed at the minimum, median and maximum of each covariate, inside the full model and compared with its linear term by likelihood ratio on one degree of freedom, the rank the spline basis adds to a model that already holds the linear term; none reached conventional significance, the smallest being $p = 0.08$ for retention volume. Spline terms were not retained: they would substantially increase the parameter count in the smaller treatment arm, and no test indicated a departure large enough to justify that cost. A smaller model was also fitted using 5 covariates selected on clinical grounds. These were oral anticoagulant use, infection at presentation, anatomical obstruction (suspected bladder-neck contracture or known urethral stricture), prior transurethral or laser prostatectomy, and age.

Collinearity among the covariates is modest. The highest variance inflation factor was 1.92, for known urethral stricture, across the 19 covariates.

S1.3. Interval estimation with fifteen clusters

Three further intervals were computed for comparison with the primary one: a bootstrap that resampled patients rather than operators; a wild cluster procedure with Rademacher weights, enumerated over the whole sign space rather than sampled from it, since 15 clusters give 32,768 sign vectors, so the p value carries no simulation error, although it remains a bootstrap approximation and not an exact test; and a cluster-robust CR2 interval with Satterthwaite degrees of freedom. The wild cluster bootstrap and the CR2 interval hold the weights fixed, because perturbing residuals cannot refit a propensity model; the operator bootstrap refits it. The fixed-weight intervals and the operator bootstrap therefore address different parts of the same question; the operator bootstrap interval is given in Table 2 and the three comparison intervals in Table S5.

Table S1. Target trial specification and how the observational analysis emulated each component.

Component	Target trial	Emulation in this cohort
Eligibility	Men with acute urinary retention in whom a first urethral catheterisation attempt has failed	The same, applied to consecutive presentations over seven years with no further criterion
Strategies	Attempt a hydrophilic guidewire, proceeding to cystostomy if it fails, versus proceed directly to cystostomy	The two strategies in routine use at the centre, neither protocolised

Component	Target trial	Emulation in this cohort
Assignment	Randomised at the moment of failure	DEPARTURE. Chosen by the attending urologist; measured differences adjusted for by overlap weighting on a propensity model, which is not a substitute for randomisation
Time zero	Randomisation	The documented failure of urethral catheterisation, identical for both arms
Outcome	Any new complication of Clavien–Dindo grade II or above within 30 days, with a secondary composite restricted to events either strategy could produce at the same grade threshold	The same two outcomes, ascertained from the record; route-specific events additionally tabulated as description
Follow-up	From time zero to 30 days	DEPARTURE. Record-based rather than active: every patient was checked for 30 days in this hospital's record and in the national personal health record, so follow-up extends beyond this institution but still misses an event that produced no reported encounter
Analysis	Intention to treat	Analysis under the strategy first attempted, retaining crossovers in their assigned arm

Components follow Hernán and Robins (American Journal of Epidemiology 2016; reference 21 of the main text). Departures from the target trial are marked in the table. No protocol fixed the analysis: the primary outcome was specified in advance but the secondary and exploratory outcomes and the analysis strategy were developed during the analysis.

No directed acyclic graph is presented: the confounding structure that the propensity model addresses is treatment selection by the attending urologist, which the assignment row states directly.

Table S2. Composition of the primary outcome among the patients who met it: highest Clavien–Dindo grade and event type, by initial strategy.

Component of the primary outcome	Direct suprapubic cystostomy	Guidewire-first
Patients with a grade ≥II event		
Total	36	7
of whom crossover patients	—	1
Highest Clavien–Dindo grade reached		
Grade II	17	7
Grade IIIa	18	0

Component of the primary outcome	Direct suprapubic cystostomy	Guidewire-first
Grade IIIb	1	0
Grade IV or V (no death occurred in either arm)	0	0
Events, counted once per patient per type		
New febrile urinary tract infection	7	4
Febrile infection already present at time zero	3	0
Visible haematuria	10	3
Catheter blockage	0	1
Transfusion	10	3
Cystostomy malposition	12	0
Loss of the cystostomy tract	6	0
Bowel injury	1	0
Reintervention within 30 days	18	0

Every row counts only the patients who met the primary outcome. Counts here are therefore no larger than the cohort-wide counts in Table 2, which include events that did not by themselves qualify a patient; catheter blockage and visible haematuria are graded I and qualify no one on their own.

A febrile infection already present at time zero did not itself qualify a patient for the primary outcome; those men entered through another new event.

A patient enters the primary outcome once, at his highest grade; the event rows count each type once per patient and therefore sum to more than the patient total.

Every grade IIIa event in the cystostomy arm was a reintervention for malposition or a lost tract, and the single grade IIIb event was the laparotomy for bowel injury.

Events occurring after hospital discharge were captured from this hospital's record and from the national personal health record, both checked for every patient over the 30 days from time zero.

Vital status at 30 days was checked for every patient in the national record; no death occurred in either arm. Organ support was not separately abstracted, so the grade IV cell rests on the absence of a recorded event.

Table S3. Balance diagnostics that overlap weighting does not fix by construction: variance ratios for the continuous covariates in the propensity model, and standardised differences for baseline variables outside it.

Variable	Before weighting	After weighting
Variance ratio, continuous covariates in the propensity model (guidewire-first over direct cystostomy)		
Age, years	1.15	1.17

Variable	Before weighting	After weighting
Body-mass index, kg/m²	0.97	0.93
Retention volume, mL	1.28	1.30
INR	6.47	1.16
Creatinine, mg/dL	1.08	1.06
Haemoglobin, g/dL	1.02	0.93
Platelets, ×10⁹/L	1.21	1.31
Standardised difference, baseline variables not entered in the propensity model		
Coronary artery disease	0.009	0.023
Chronic kidney disease	−0.010	−0.009
Any antithrombotic agent	0.267	0.000

Overlap weighting equalises the weighted mean of every covariate entered in the model, so the standardised difference of those covariates is zero after weighting by construction and says nothing about the model. The two diagnostics here carry no such guarantee: a variance ratio can fail even for a modelled covariate, and the variables in the lower block never entered the model.

A variance ratio between 0.5 and 2.0 is conventionally regarded as acceptable (Austin, Statistics in Medicine 2009; reference 25 of the main text). After weighting, 0 of the 7 continuous covariates fell outside that range.

Standardised differences are signed as guidewire-first minus direct suprapubic cystostomy. Any antithrombotic agent is the union of the oral anticoagulant and antiplatelet terms that are in the model, so its weighted difference is also algebraic; coronary artery disease and chronic kidney disease are independent of the model.

INR, international normalised ratio.

Table S4. Strategy use by operator.

Operator	Patients	Guidewire-first
Operator 1	27	37.0% (10/27)
Operator 2	36	25.0% (9/36)
Operator 3	12	16.7% (2/12)
Operator 4	31	12.9% (4/31)
Operator 5	33	36.4% (12/33)
Operator 6	21	57.1% (12/21)

Operator	Patients	Guidewire-first
Operator 7	18	22.2% (4/18)
Operator 8	14	28.6% (4/14)
Operator 9	26	23.1% (6/26)
Operator 10	24	20.8% (5/24)
Operator 11	32	40.6% (13/32)
Operator 12	17	41.2% (7/17)
Operator 13	25	32.0% (8/25)
Operator 14	16	18.8% (3/16)
Operator 15	20	35.0% (7/20)

Operators are numbered arbitrarily and are not identifiable.

Strategy use varied across operators; the primary analysis therefore resamples operators rather than patients when forming intervals.

Table S5. Sensitivity analyses of overall strategy-associated complications and of shared complications under alternative estimators and propensity specifications.

Analysis	Absolute risk difference, percentage points (95% CI)
Overall strategy-associated complications (primary outcome): new grade ≥II event, either route	
Stabilised inverse-probability weights	−11.7 (−17.1 to −4.1)
Doubly robust estimator	−10.2 (−16.1 to −1.3)
Propensity model including year of procedure	−12.2 (−18.9 to −5.4)
Propensity model including operator fixed effects	−14.1 (−20.2 to −7.2)
Clinically restricted propensity model (5 covariates)	−11.9 (−17.8 to −5.3)
Bootstrap resampling patients rather than operators	−12.2 (−19.2 to −5.7)
Wild cluster bootstrap over operators	−12.2 (−19.7 to −4.8)
Cluster-robust CR2 interval, Satterthwaite degrees of freedom	−12.2 (−19.5 to −5.0)

Analysis	Absolute risk difference, percentage points (95% CI)
Shared complications (secondary outcome): new grade ≥II with a shared event	
Stabilised inverse-probability weights	−3.9 (−10.0 to 5.4)
Doubly robust estimator	−2.1 (−8.4 to 9.1)
Propensity model including year of procedure	−4.1 (−10.9 to 3.9)
Propensity model including operator fixed effects	−6.5 (−13.4 to 2.5)
Clinically restricted propensity model (5 covariates)	−3.3 (−10.2 to 4.4)
Bootstrap resampling patients rather than operators	−4.2 (−10.3 to 1.6)
Wild cluster bootstrap over operators	−4.2 (−12.5 to 4.1)
Cluster-robust CR2 interval, Satterthwaite degrees of freedom	−4.2 (−12.4 to 4.0)

Each row re-estimates the outcome named in the block heading under a different estimator or propensity specification. The primary analysis of each outcome is given in Table 2.

Overlap weighting targets the overlap population. Stabilised inverse-probability and doubly robust estimators target the whole cohort and are therefore adjacent rather than identical estimands.

The restricted model holds oral anticoagulant use, infection at presentation, an anatomical obstruction, a prior transurethral or laser prostatectomy, and age. Spline terms were not retained in any model because they would raise the parameter count in the smaller arm; the linearity checks are reported in Supplementary Methods S1.2.

The wild cluster bootstrap and the CR2 interval hold the propensity weights fixed and therefore account for the clustering over 15 operators rather than for the estimation of the weights; the operator bootstrap used for the primary analysis refits the propensity model within each resample and accounts for both.

CI, confidence interval.

Table S6. Baseline characteristics associated with guidewire failure, exploratory.

Characteristic	Failures / patients with it	Failures / patients without it	Odds ratio (95% CI)
Suspected bladder-neck contracture	4 / 6	12 / 100	OR 14.67 (95% CI 2.42 to 88.85, Woolf; Fisher exact p = 0.005)
Known urethral stricture	7 / 14	9 / 92	OR 9.22 (95% CI 2.63 to 32.29, Woolf; Fisher exact p < 0.001)
Prior TUR-P or HoLEP	6 / 14	10 / 92	OR 6.15 (95% CI 1.77 to 21.37, Woolf; Fisher exact p = 0.007)

Characteristic	Failures / patients with it	Failures / patients without it	Odds ratio (95% CI)
Prior difficult catheterisation	8 / 21	8 / 85	OR 5.92 (95% CI 1.89 to 18.57, Woolf; Fisher exact p = 0.003)
Prior urethral surgery	3 / 11	13 / 95	OR 2.37 (95% CI 0.55 to 10.09, Woolf; Fisher exact p = 0.37)

Exploratory and unadjusted. The arm holds 106 patients and 16 failures, so a multivariable model would be unstable and none was fitted.

These five characteristics were the only ones examined; no wider screen was performed. Odds ratios are the sample (Woolf) estimate with its Woolf interval and the Fisher exact p value, and are reported to describe this cohort rather than to support a triage rule.

TUR-P, transurethral resection of the prostate; HoLEP, holmium laser enucleation of the prostate.
